# Population Genetic Structure of *Aedes (Stegomyia) aegypti* (L.) at a Micro-Spatial Scale in Thailand: Implications for a Dengue Suppression Strategy

**DOI:** 10.1371/journal.pntd.0001913

**Published:** 2013-01-10

**Authors:** Phanthip Olanratmanee, Pattamaporn Kittayapong, Chitti Chansang, Ary A. Hoffmann, Andrew R. Weeks, Nancy M. Endersby

**Affiliations:** 1 Centre of Excellence for Vectors and Vector-Borne Diseases, Faculty of Science, Mahidol University at Salaya, Nakhon Pathom, Thailand; 2 Department of Biology, Faculty of Science, Mahidol University, Bangkok, Thailand; 3 National Institute of Health, Department of Medical Sciences, Ministry of Public Health, Nonthaburi, Thailand; 4 Pest and Disease Vector Control Group, Bio 21 Institute, Department of Genetics, University of Melbourne, Victoria, Australia; Institute of Tropical Medicine (NEKKEN), Japan

## Abstract

**Background:**

The genetic population structure of *Aedes (Stegomyia) aegypti* (L.), the main vector of dengue virus, is being investigated in areas where a novel dengue suppression program is to be implemented. The aim of the program is to release and establish mosquito populations with impaired virus transmission capabilities. To model effects of the release and devise protocols for its implementation, information about the genetic structure of populations at a range of spatial scales is required.

**Methodology/Principal Findings:**

This study investigates a potential release site in the Hua Sam Rong Subdistrict of Plaeng Yao District, Chachoengsao Province, in eastern Thailand which comprises a complex of five villages within a 10 km radius. *Aedes aegypti* resting indoors was sampled at four different times of year from houses within the five villages. Genetic markers were used to screen the mosquitoes: two Exon Primed Intron Crossing (EPIC) markers and five microsatellite markers. The raw allele size was determined using several statistical software packages to analyze the population structure of the mosquito. Estimates of effective population size for each village were low, but there was no evidence of genetic isolation by geographic distance.

**Conclusions:**

The presence of temporary genetic structure is possibly caused by genetic drift due to large contributions of adults from a few breeding containers. This suggests that the introduction of mosquitoes into an area needs to proceed through multiple releases and targeting of sites where mosquitoes are emerging in large numbers.

## Author Summary

Knowledge about population structure of *Aedes (Stegomyia) aegypti* (L.), the main vector of dengue virus, is vital in designing dengue suppression programs. To design a release of dengue resistant mosquitoes, we require information about population structure at a range of spatial scales. This study looked at a small-scale complex of five villages at four sampling periods and mosquitoes from individual houses within the five villages. Genetic markers were used to screen the field-collected mosquitoes in order to characterize population genetic structure. Our results indicated that the effective population size of *Ae. aegypti* in each village was low. Genetic structure was apparent at local spatial scales, but there was no evidence of genetic isolation by geographic distance. The temporary genetic structure observed may be caused by related individuals emerging from a few productive containers in each village. Our findings could be applied in an effective dengue suppression program by planning multiple releases of dengue resistant mosquitoes in targeted households of each village where large numbers of adult mosquitoes have emerged from identified productive breeding containers instead of making a release in one area and expecting rapid spread throughout the complex.

## Introduction

Though *Aedes (Stegomyia) aegypti* (L.) may have been present in Thailand in the 19^th^ century, its spread from major urban centers and commercial transport routes to rural villages is thought to have occurred in the last 50 years [1]. Patterns of day to day movement and distances travelled by *Ae*. *aegypti* at different spatial scales and at different densities of human settlement determine the nature of spread of dengue virus and are also important in modeling the effects of potential control strategies.

Artificial infection of *Ae. aegypti* with strains of the bacterium, *Wolbachia pipientis*, has been shown to reduce vector competence of the mosquito for dengue virus [2]. Field releases of *Wolbachia*-infected *Ae*. *aegypti* in northern Queensland, Australia have demonstrated the feasibility of spreading a *Wolbachia* infection through the wild population in a localised area [3]. Future studies, in countries such as Thailand where dengue is endemic, will look at the effect of *Wolbachia* on dengue suppression and, if successful, will be adopted as an area-wide dengue control strategy.

Detailed knowledge of population genetic structure can be translated into practical information for designing the logistics of a field release of *Wolbachia*-infected mosquitoes (i.e. how many mosquitoes to release, over what sized area and at what time of year). It is already known that collections of *Ae*. *aegypti* taken from four widely spaced samples in Chiang Mai Province in Thailand were highly differentiated (*F*_ST_ = +0.185, *P<*10^−4^) when five microsatellite markers were employed [4]. Genetic structure at distances of less than 25 km and also between samples taken more than 100 km apart has also been found in Thailand using variation in a region of the NADH dehydrogenase subunit 4 mitochondrial DNA gene (ND4) [1]. Another study using 13 microsatellite loci to look at population genetics of *Ae. aegypti* in mainland Southeast Asian countries revealed genetic structure at all spatial scales including those at a distance of less than 500 m [5]. The short-range nature of dispersal of *Ae*. *aegypti* was demonstrated by mark-release-recapture experiments in which a majority of mosquitoes did not move from their release house or the one adjacent, while those released outdoors moved a maximum of 512 m [6].

The current study aims to collect population genetic information from within a complex of five villages in Thailand contained within an area of 314 km^2^. The village complex is a potential release site for *Wolbachia*-infected mosquitoes that could resist dengue virus infection, so it is important to find out what patterns of mosquito movement and population size occur within and between these specific villages at different times of the year. The implications of the patterns identified will be factored into strategies for release and establishment of *Wolbachia*-mediated dengue resistant mosquitoes. These mosquitoes are intended to replace the natural population of *Ae*. *aegypti* at a target release site and then be spread throughout the country thereby reducing the opportunity for transmission of dengue virus in Thailand.

## Materials and Methods

### Sampling

**Figure 1:**
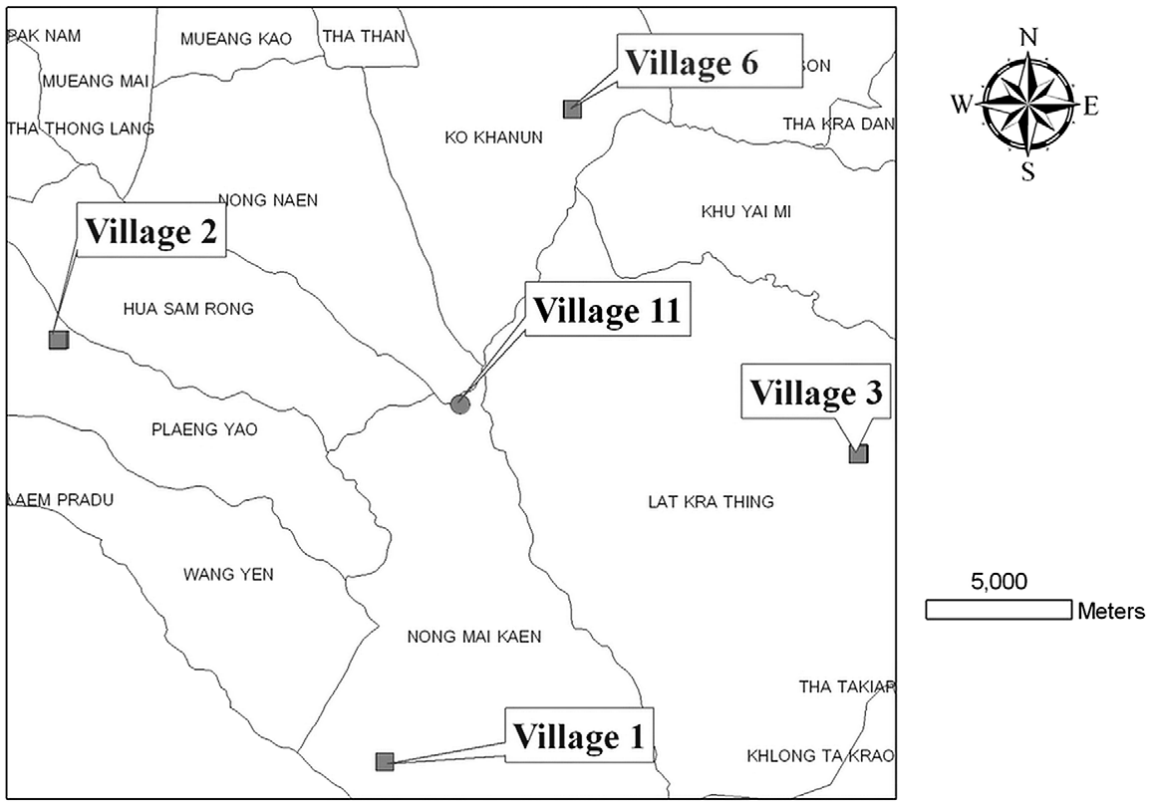
Complex of five villages in Hua Sam Rong Subdistrict of Plaeng Yao District, Chachoengsao Province, eastern Thailand, from which samples of *Aedes (Stegomyia) aegypti* (L.) were taken from December 2007 to September 2008.

Samples of adult *Ae*. *aegypti* mosquitoes were collected from a complex of five villages: one central village (Village 11) and four outer villages (Villages 1, 2, 3, 6) within a radius of 10 km in the Hua Sam Rong Subdistrict of Plaeng Yao District, Chachoengsao Province, in eastern Thailand (Figure 1). These five villages are located in semi-rural and rural areas of the province. Households in each village are mostly distributed in a small cluster of 5–10 houses, except for Village 6 which is mostly composed of individual houses shaded by vegetation. Village 11 is located in the foothills of the mountains. Village 2 has a man-made canal for irrigation and most of the land was used as rice paddy fields while another part of this village is considered to be a semi-rural commercial area. Villages 1, 3 and 11 are mainly surrounded by rubber plantations. All villages are geographically separated by rice paddy field, rubber plantations and unused land.

Adult mosquitoes were collected using vacuum aspirators from houses after receiving permission from the head of the household. These villages are being studied as a potential release site for dengue resistant mosquitoes. Four samplings were made from individual houses in all five villages, i.e., one center village and four other villages within a radius of at least 10 km, at three-month intervals in December 2007 (cool-dry season), March 2008 (hot-dry season), June 2008 (wet season) and September 2008 (wet season) (Table 1). The sample location of individual mosquitoes in the five-village complex was recorded (data not shown).

**Table 1:** Population characteristics of *Aedes (Stegomyia) aegypti* (L.) sampled from five villages in Chachoengsao Province, eastern Thailand (*r* = allelic richness, *F*_IS_ = inbreeding coefficient, *H*_E_ = expected heterozygosity, *H*_O_ = observed heterozygosity, HW-*P* = Hardy Weinberg *P* value, *significant after correction for multiple comparisons using False Discovery Rate procedures [12], N/S = no spatial autocorrelation at distances tested), Relatedness estimator [22], GGD = geographic distance.

Sample site	Date	N	*R*	*F*_IS_	*H*_E_	*H*_O_	HW-*P*	Spatial autocorrelation		Relatedness vs GGD
								Distance (m)	*P*	*R^2^*
Village 11	Dec 2007	47	4.128	0.126	0.534	0.473	0.001*	500	0.032	0.0019
Village 11	Mar 2008	30	3.848	0.107	0.508	0.462	0.023	N/S	N/S	0.0003
Village 11	Jun 2008	30	3.719	0.010	0.538	0.542	0.430	200	0.010	0.0064
Village 11	Sep 2008	31	3.321	0.130	0.530	0.470	0.007*	1900	0.010	0.0003
Village 1	Dec 2007	50	3.487	0.021	0.453	0.448	0.330	100	0.010	0.0163
Village 1	Mar 2008	32	3.820	0.010	0.527	0.530	0.419	N/S	N/S	0.0001
Village 1	Jun 2008	32	3.701	0.144	0.474	0.414	0.004*	N/S	N/S	0.0004
Village 1	Sep 2008	31	3.214	0.060	0.533	0.510	0.119	1300	0.018	0.00002
Village 2	Dec 2007	40	3.480	0.085	0.526	0.488	0.038	N/S	N/S	0.0001
Village 2	Mar 2008	32	3.254	0.063	0.480	0.458	0.122	N/S	N/S	0.0002
Village 2	Jun 2008	32	3.795	0.101	0.544	0.498	0.026	900	0.006	0.0001
Village 2	Sep 2008	32	3.899	0.052	0.551	0.531	0.151	550, 850	0.006, 0.030	0.0034
Village 3	Dec 2007	38	3.432	0.110	0.543	0.491	0.013	100, 400	0.002, 0.001	0.0121
Village 3	Mar 2008	30	3.940	0.051	0.551	0.533	0.175	N/S	N/S	0.0094
Village 3	Sep 2008	31	3.879	0.034	0.563	0.554	0.251	N/S	N/S	0.00003
Village 6	Dec 2007	48	3.553	0.016	0.499	0.496	0.355	100, 1000	0.004, 0.003	0.0005
Village 6	Mar 2008	30	3.263	−0.043	0.456	0.483	0.791	N/S	N/S	0.0026
Village 6	Sep 2008	33	3.952	0.070	0.525	0.497	0.076	100	0.034	0.001

### Ethical considerations

This study was approved by the Institutional Review Board of Mahidol University at Salaya, Nakhon Pathom, Thailand under the project entitled: Control of the Dengue Vector, *Aedes aegypti*, by Population Replacement Using *Wolbachia*-Infected Mosquito Strain” (COA. No. MU-IRB 2011/227).

### DNA Extraction and PCR preparation

Genomic DNA of individual mosquitoes was extracted using the Holmes and Bonner method [7]. Microsatellite PCR amplification was made in a volume of 10 µl: 2 µl of genomic DNA, 0.03 µM forward primer end-labelled with [γ^33^P]-ATP, 0.4 µM reverse primer, 2.0 mM MgCl_2_, 0.1 mM dNTPs, 0.5 mg/ml purified bovine serum albumin (New England Biolabs, Ipswich, MA), 1 µl of 10× PCR amplification buffer, and 0.4 units of *Taq* polymerase (New England Biolabs, Ipswich, MA).

### Molecular screening

Seven markers were used to screen *Ae. aegypti* from the five villages due to their consistent performance under our laboratory conditions: two Exon Primed Intron Crossing (EPIC) markers; Rps20b, RpL30a [8] and five microsatellite markers; AC1, AG5 [9], BbA10, BbH08 [10] and Gyp8 [8].

### Analyses

A global estimate (with 95% confidence limits) [11] and population pairwise measures of *F*_ST_ with significance determined using permutations were obtained using FSTAT version 2.9.3 [12] to look for population structure within the data. Allelic richness per population averaged over loci and Weir and Cockerham's [11] measure of *F*_IS_ were also estimated with FSTAT. Correction for multiple comparisons was made using False Discovery Rate procedures [13].

Observed (*H*_O_) and expected (*H*_E_) heterozygosity were estimated using GenAlEx version 6 [14] and deviations from Hardy-Weinberg equilibrium were tested using Genepop version 3.4 [15] to check for technical problems with the markers or unusual population processes. A Mantel test of the linearized *F*_ST_ transformation [*F*_ST_/(1−*F*_ST_)] with the natural log of geographical distance [16] was made with POPTOOLS version 2.6 [5] to investigate whether gene flow was largely restricted by geographic distance (isolation by distance). Significance of Mantel tests was determined by permutation (10,000 randomizations).

Analysis of molecular variation (AMOVA) was undertaken in Arlequin v3.11 [17] using pairwise *F*_ST_ as the distance measure, with 10,000 permutations and missing data for loci set at 10%. The model for analysis partitioned variation among groups (villages), among populations within groups (temporal samples from each village) and within samples.

A factorial correspondence analysis was run in Genetix v4.03 [18] to summarize patterns of genetic differentiation between the populations sampled. We plotted the first model underlying factors that explain the majority of the variation in the multi-locus genotypes. A second analysis to estimate the number of populations within the sample data was made with Geneland 3.1.4 [19], a program which takes both spatial and genetic data into account. Clusters are formed so that each population is in approximate Hardy-Weinberg equilibrium with linkage equilibrium between loci (HWLE). The model was run for the data from each 3-monthly sampling occasion across the five-village complex and for each village over the complete sampling period. The “correlated allele frequency” option was chosen for the multiple sampling events at one site. For all other runs across the geographic area, we used the “allele frequencies uncorrelated” option. 500,000 iterations were applied with a thinning factor of 100. Five independent runs were made for each data set with a burn-in of 200 (*100). The number of pixels in the spatial design was set at 50*50. If estimates of *K* were not consistent, then the estimate with the highest average posterior probability was used.

The temporal method of Waples [20] in the program NeEstimator [21] was applied to estimate effective population size (*N*_e_) for mosquitoes in the five village sample. Approximate generation time of one month was assumed for this analysis. The initial sample from each village (Gen 0 – Dec 2007) was compared with the final sample from the same village (Gen 9 – Sep 2008). Waples' [20] formula uses variation in allele frequencies across generations to estimate the harmonic mean of each generation's *N*_e_. Using widely spaced temporal samples to estimate *N*_e_ reduces bias associated with sampling in species with overlapping generations [21].

Spatial analysis looks for patches of genetic correlation by comparing pairs of individuals within distance classes. To look at spatial autocorrelation within a village, the autocorrelation co-efficient *r* was estimated for samples in 50–100 m size classes for each village on each sampling occasion using GenAlEx [14]. Maximum distance between individuals sampled in a village was 3,300 m. Statistical significance of *r* was tested in GenAlEx by random permutation of individuals around each geographic location to create a distribution under the assumption of no spatial structure.

Ritland's [23] Relatedness Index was estimated for pairwise sample comparisons from each village and for each season using GenAlEx. These pairwise comparisons of relatedness were plotted against pairwise geographic distance (m) to look for spatial trends of relatedness within villages.

## Results

### Basic population genetic parameters

Null alleles over all loci were suspected in populations from Village 3 and Village 6 in June 2008 due to a DNA amplification problem, so results from these samples were not included in the analysis. Estimates of allelic richness ranged from 3.21 (Village 1 – Sep 2008) to 4.13 (Village 11 – Dec 2007), but there were no apparent seasonal or spatial trends (Table 1). Allele frequencies from mosquitoes collected in Village 11 in Dec 2007 were not in Hardy-Weinberg equilibrium even after correction for multiple comparisons. The measure of inbreeding, *F*_IS_, ranged from 0.01 (Village 11 – Jun 2008, Village 1 – Mar 2008) to 0.14 (Village 1 Jun 2008) (Table 1). After adjustment for multiple comparisons, three of the estimates of *F*_IS_ were significant: Village 11 (Dec 2007 and Sep 2008) and Village 1 (June 2008) (FDR adjusted *P*-values: 0.009, 0.0402 and 0.0351 respectively for classical one step method). Application of a further FDR graphically sharpened method of correction showed significant *P*-values for Village 3 (Dec 2007), Village 2 (Dec 2007, June 2008) and Village 11 (Mar 2008) (FDR adjusted *P*-values: 0.016, 0.027, 0.021, 0.021), indicating that inbreeding is a common occurrence.

### Population differentiation

The estimate of *F*_ST_ over all populations was 0.037 (0.022–0.046; 99% confidence intervals) indicating that there was some genetic structure in the dataset as a whole (i.e. *F*_ST_>0). The AMOVA was significant for each of the variance components at each level of the hierarchical model: among villages (percentage of variation = 1.16%, Va = 0.02, SS = 40.02, df = 4, *P*<0.05), among temporal samples within villages (percentage of variation = 2.41%, Vb = 0.04, SS = 60.89, df = 13, *P*<0.001) and within sampling occasions (percentage of variation = 96.42%, Vc = 1.72, SS = 2128.93, df = 1240, *P*<0.001), suggesting both spatial and temporal differentiation of mosquito populations. There was no significant correlation between genetic distance and geographic distance (Mantel *r* = 0.019, *P* = 0.359) at the small spatial scale of this study.

### Spatial structure at the village level

Analysis of genetic and spatial structure with Geneland showed that the genotypes for the December 2007 sample fall into two clusters, one comprising the samples from the central village (11) and the second cluster containing the samples from the four surrounding villages (data not shown). For each of the other sampling occasions within a season (March, June and September 2008), the complete dataset comprised only one cluster (*K* = 1).

### Temporal changes in structure within villages

**Figure 2:**
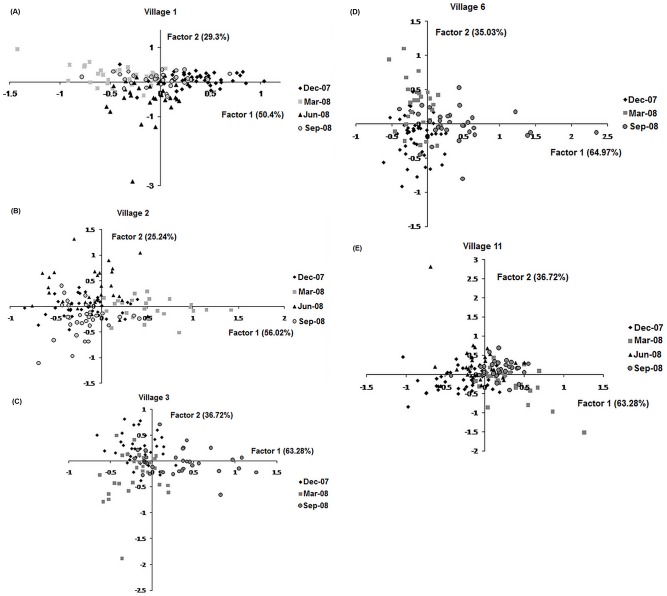
Temporal differences in population structure in *Aedes (Stegomyia) aegypti* (L.) in a complex of five eastern Thai villages, identified with Factorial Correspondence Analysis ((A) Village 1; (B) Village 2; (C) Village 3; (D) Village 6 and (E) Village 11).

Factorial Correspondence Analysis revealed some seasonal differences within village samples (Figure 2 shows seasonal differences within each village). This was reflected in some of the pairwise estimates of *F*_ST_ (Table 2). The number of populations per site over all sampling periods estimated by Geneland showed that *K* = 2 for samples from Village 11 and 3 and *K* = 3 for Village 1, 2 and 6. These clusters appear to be related mainly to temporal differences in the absence of spatial structure.

**Table 2:** Pairwise population comparisons of *Aedes (Stegomyia) aegypti* (L.) from five villages in Chachoengsao Province, eastern Thailand.

	V11Dec07	V11Mar08	V11Jun08	V11Sep08	V1Dec07	V1Mar08	V1Jun08	V1Sep08	V2Dec07	V2Mar08	V2Jun08	V2Sep08	V3Dec07	V3Mar08	V3Sep08	V6Dec07	V6Mar08	V6Sep08
V11Dec07	0	0.0165	0.0081	0.0291	**0.1189**	0.0179	0.0392	**0.0670**	**0.0562**	**0.0684**	**0.0461**	**0.0299**	**0.0712**	**0.0350**	**0.0408**	**0.0723**	**0.0704**	**0.0275**
V11Mar08	0	0	0.0111	0.0039	**0.0567**	**0.0196**	0.0124	**0.0384**	**0.0326**	**0.0751**	0.0224	0.0236	**0.0402**	0.0260	**0.0286**	**0.0461**	**0.0718**	0.0124
V11Jun08	0	0	0	0.0135	**0.0862**	0.0051	0.0255	0.0249	**0.0248**	**0.0567**	**0.0236**	0.0288	**0.0500**	0.0263	0.0165	**0.0504**	**0.0553**	0.0060
V11Sep08	0	0	0	0	**0.0759**	**0.0278**	0.0410	**0.0304**	**0.0380**	**0.0732**	**0.0260**	**0.0397**	**0.0477**	**0.0364**	**0.0386**	**0.0594**	**0.0811**	0.0237
V1Dec07	14.63	14.63	14.63	14.63	0	**0.0796**	**0.0563**	**0.0323**	**0.0337**	**0.1185**	**0.0351**	**0.0557**	**0.0406**	**0.0395**	**0.0531**	**0.0492**	**0.1087**	**0.0441**
V1Mar08	14.63	14.63	14.63	14.63	0	0	0.0170	**0.0254**	**0.0335**	**0.0416**	0.0181	0.0149	**0.0445**	0.0137	0.0147	**0.0472**	**0.0386**	−0.0054
V1Jun08	14.63	14.63	14.63	14.63	0	0	0	0.0352	0.0223	**0.0689**	0.0071	0.0224	0.0453	0.0295	0.0285	0.0425	0.0528	0.0039
V1Sep08	14.63	14.63	14.63	14.63	0	0	0	0	0.0062	**0.0564**	−0.0010	0.0280	0.0148	0.0088	0.0019	**0.0318**	**0.0562**	0.0024
V2Dec07	13.35	13.35	13.35	13.35	18.66	18.66	18.66	18.66	0	**0.0870**	−0.0009	0.0135	**0.0255**	**0.0163**	0.0201	**0.0402**	**0.0810**	0.0122
V2Mar08	13.35	13.35	13.35	13.35	18.66	18.66	18.66	18.66	0	0	**0.0493**	**0.0812**	**0.0397**	0.0298	**0.0563**	0.0183	−0.0112	0.0326
V2Jun08	13.35	13.35	13.35	13.35	18.66	18.66	18.66	18.66	0	0	0	0.014	0.0077	−0.0013	0.0112	0.0215	**0.0443**	−0.0016
V2Sep08	13.35	13.35	13.35	13.35	18.66	18.66	18.66	18.66	0	0	0	0	**0.0329**	0.0124	0.0089	**0.0498**	**0.0800**	0.0120
V3Dec07	13.57	13.57	13.57	13.57	19.30	19.30	19.30	19.30	26.92	26.92	26.92	26.92	0	0.0009	0.0203	0.0104	**0.0418**	0.0171
V3Mar08	13.57	13.57	13.57	13.57	19.30	19.30	19.30	19.30	26.92	26.92	26.92	26.92	0	0	0.0093	0.0113	0.0299	0.0020
V3Sep08	13.57	13.57	13.57	13.57	19.30	19.30	19.30	19.30	26.92	26.92	26.92	26.92	0	0	0	**0.0413**	**0.0559**	0.0014
V6Dec07	7.80	7.80	7.80	7.80	22.43	22.43	22.43	22.43	18.69	18.69	18.69	18.69	14.75	14.75	14.75	0	0.0142	0.0246
V6Mar08	7.80	7.80	7.80	7.80	22.43	22.43	22.43	22.43	18.69	18.69	18.69	18.69	14.75	14.75	14.75	0	0	0.0278
V6Sep08	7.80	7.80	7.80	7.80	22.43	22.43	22.43	22.43	18.69	18.69	18.69	18.69	14.75	14.75	14.75	0	0	0

Upper diagonal shows pairwise *F*_ST_ estimates, lower diagonal shows pairwise geographic distance (km). Significance of *F*_ST_ estimates (obtained by 15300 permutations) at the indicative adjusted nominal level (5%) of *P*<0.000327 is indicated by bold type.

### Effective population size

Estimates of effective population size (*N*_e_) in the five villages were low and ranged from 92.7 to 221.5 (Table 3). Approximate 95% confidence intervals were widest for Village 2 (66.5–4830.0).

**Table 3:** Estimates of effective population size (*N*_e_) of *Aedes (Stegomyia) aegypti* (L.) in five villages in Thailand based on Waples' [20] method using temporal differences in allele frequency across 9 generations.

Village	*N*_e_	Approx 95% confidence intervals	
		lower	Upper
Village 11	160.6	59.7	623.4
Village 1	92.7	37.9	233.7
Village 2	221.5	66.5	4830.0
Village 3	150.4	51.8	729.4
Village 6	205.9	71.8	1247.2

### House scale spatial autocorrelation

The spatial autocorrelation analyses indicated significant genetic correlations between individuals in a range of distance classes based on the nonparametric permutational tests employed by Smouse & Peakall [24]. If a more conservative approach is taken and correction for multiple comparisons is made using either the Bonferroni method [25] or the False Discovery Rate procedure [16], then none of the correlations is significant (Table 1). The correlations follow no specific trend which is consistent with the lack of a significant relationship between genetic and geographic distance suggested by the Mantel test using between village distances; however 100 m is the most commonly significant size class (pre-correction), suggesting some association of genotypes at very short distances. Moreover, regressions of Ritland's [23] Relatedness Index with geographic distance for pairwise sample comparisons from each village and for each season tended to be negative (13 out of 18 cases, one tailed Sign test, *P* = 0.048), suggesting a decrease in relatedness with increasing distance although R^2^ values for the negative relationships were low (0.0001 to 0.012) (Table 1). The highest *R* ^2^ values came from Villages 1 and 3 in December 2007.

## Discussion

The Hua Sam Rong Subdistrict of Plaeng Yao District, Chachoengsao Province, in eastern Thailand is a potential release site for mosquitoes that have a reduced capacity to transmit dengue virus. Information about mosquito movement within this Subdistrict in Village 6 has been collected in mark-release-recapture studies [6]. Genetic methods of assessing mosquito population structure and movement have been employed in this study to add to the current knowledge and provide detailed information about within- and between-village gene flow which will be pertinent to the release.

In general terms, there was evidence for genetic structure (spatial and temporal) in the dataset as a whole, though it did not follow a pattern of genetic isolation by geographic distance. Our results confirm the finding of Hlaing et al. [5] which reported low but significant genetic structure at all spatial scales within mainland Southeast Asia. Seasonal shifts in allele frequency in the villages may occur when there is a particularly productive water container which leads to a local explosion in mosquito numbers and a genetic bottleneck has changed allele frequencies within this container. In addition, it may occur when a mosquito outbreak occurs in one place and spreads across villages. Patterns of genetic distance would be different if the effect of either of these scenarios predominates. The first scenario would be expected to produce high levels of genetic distance between villages within a season, within a village between seasons and between villages across seasons. In the second scenario in which a sweep of mosquitoes moves through the village complex, high genetic distances within villages between seasons would be observed. Genetic distance between villages would be low, both within and between seasons if such sweeps were common. Intermediate or mixed models could also apply, but it is useful, in the first instance, to investigate the data in terms of these extremes.

Based on the clusters assigned by Geneland for each collection month (March, June and September 2008), there was no genetic differentiation of *Ae*. *aegypti* between villages which would be expected under the second scenario, i.e. a widespread outbreak and genetic homogenization. Based on estimates of pairwise *F*_ST_, samples from Villages 11, 1 and 2 show within-village seasonal differences which could also indicate sweeps of mosquitoes moving through at least part of the village complex in different seasons. The assignment of samples from Village 11 in December 2007 to a different Geneland cluster from all other villages suggests a local outbreak effect as proposed in the first scenario. Samples from Village 1 in December 2007 are differentiated from all others according to pairwise comparisons of *F*_ST_ which is evidence of local structure which Geneland did not characterize. Both Villages 3 and 6, show no seasonal within-village patterns of differentiation in pairwise *F*_ST_. However, when spatial characteristics are taken into account by Geneland, samples from Village 3 are assigned to two seasonal-based clusters. Similarly, samples from Village 6 comprise three clusters based on spatial and seasonal characteristics.

Larval abundance in the Subdistrict is highest in the wet season (May–October) and dengue transmission is also most common at this time [26]. Abundance of larvae has been found to vary greatly over short distances [26] and may influence population structure as described in the first scenario. Major control efforts in particular areas should also be taken into account as they could cause local extinction and recolonisation from elsewhere with potential to change allele frequencies.

Larval abundance in houses in Thai villages can be high; for instance Strickman and Kittayapong [26] estimate 49 to 173 larvae per house. This would seem to indicate the potential for large population sizes maintaining abundant genetic variation, in contrast to the genetic structure and relatively low N_e_ estimates obtained here. However it is known that a majority of mosquito larvae do not become adults [27]. For example, Dye (1984) predicted that an average of 19.1% of larvae survive to the mid-pupal stage in populations of *Ae*. *aegypti* from Bangkok [28]. Density of adult *Ae*. *aegypti* in Thailand has been described as low [29]; intensive collections in 100 houses from four locations yielded only 764 mosquitoes [30]. Mating success of *Ae*. *aegypti* has also been estimated as <100% in the field, for example, female insemination rates in 9 m^3^ field cages were 65–85% [29]. Each of these factors point to the potential for the effective population size to be low compared with the census size.

The temporal method for estimation of *N*_e_ [11], which compares observed changes in allele frequency to those expected under drift alone, can be biased (over or underestimated depending on sampling method) in species such as *Ae*. *aegypti*, which have overlapping generations. However, the bias can be reduced by sampling consecutive age cohorts, an option not possible in this study, or by sampling over long time intervals [22]. The sampling interval of approximately nine generations in this study should have been adequate to minimize bias as opportunity for genetic drift is increased [22] so a low *N*_e_ appears realistic. In this case, estimates of N_e_ are low and are indicative of low genetic variability (also supported by low estimates of allelic richness) as well as spatial isolation. This means that release of *Wolbachia*-infected mosquitoes should occur within each village of the complex rather than making a release in one area and expecting rapid spread throughout the complex.

The disjunctive nature of population structure over small and large distances has been attributed to transport of *Ae*. *aegypti* by humans [5], [31]. Movement in the Subdistrict is intensive between some villages in the complex. Village 2 is the main commercial center of the Hua Samrong Subdistrict and contains a school which attracts students from other nearby villages [26]. None of the villages has running water and people living in Village 6 import drinking water from other areas because village wells have become saline [26]. As a consequence, large volumes of water are stored in Village 6 and may explain the high abundance of mosquito larvae found in this village compared with nearby villages in a container survey in 1990–1991 [26]. Our estimates of effective population size (*N*_e_) showed Villages 2 and 6 as having the highest numbers, though 95% confidence intervals overlapped with estimates for the other villages.

What information do these findings give us to assist with release and establishment of the dengue resistant strain of *Ae*. *aegypti*? Initial releases will depend on movement patterns at the local spatial scale. In the five-village complex we found weak and haphazard patterns of spatial autocorrelation suggesting that, also at this scale, natural dispersal may be influenced by human transport of *Ae*. *aegypti*. In combination with short distance natural dispersal limited mainly to within a house or an adjacent house, multiple intensive releases are envisaged for putting the dengue resistant mosquitoes into a village complex in Thailand. With this type of population structure, successful *Wolbachia* invasions may depend on fine scale monitoring of infection frequencies, so that strategies can be implemented locally to counter areas where uninfected individuals may persist. Because villages form relatively isolated units, it is also possible that any evolutionary changes in the mosquito nuclear genome or viral genome in response to *Wolbachia* may remain relatively contained.
